# Impact of game jam learning about cultural safety in Colombian medical education: a randomised controlled trial

**DOI:** 10.1186/s12909-021-02545-7

**Published:** 2021-02-25

**Authors:** Juan Pimentel, Anne Cockcroft, Neil Andersson

**Affiliations:** 1 CIET-PRAM, Department of Family Medicine, McGill University, 5858 Chemin de la Côte-des-Neiges 3rd Floor, Suite 300, Montreal, Quebec, H3S 1Z1 Canada; 2 Facultad de Medicina, Universidad de La Sabana, Campus Universitario puente del común, Chía, Colombia CP 250001; 3 Escuela de Medicina y Ciencias de la Salud, Universidad del Rosario, Carrera 24 # 63, C 69 Bogotá, Colombia; 4 Centro de Investigación de Enfermedades Tropicales (CIET), Universidad Autónoma de Guerrero, Calle Pino s/n Colonia El Roble, 39640 Acapulco, Guerrero Mexico

**Keywords:** Game jam, Serious games, Co-design, Cultural safety, Medical education, Colombia

## Abstract

**Background:**

Cultural safety, whereby health professionals respect and promote the cultural identity of patients, could reduce intercultural tensions that hinder patient access to effective health services in Colombia. Game jams are participatory events to create educational games, a potentially engaging learning environment for Millennial medical students. We set out to determine whether medical student participation in a game jam on cultural safety is more effective than more conventional education in changing self-reported intended patient-oriented behavior and confidence in transcultural skills.

**Methods:**

We conducted a parallel-group, two-arm randomized controlled trial with 1:1 allocation. Colombian medical students and medical interns at University of *La Sabana* participated in the trial. The intervention was a game jam to create an educational game on cultural safety, and the reference was a standard lesson plus an interactive workshop on cultural safety. Both sessions lasted eight hours. Stratified randomization allocated the participants to the intervention and control groups, with masked allocation until commencement.

**Results:**

531 students completed the baseline survey, 347 completed the survey immediately after the intervention, and 336 completed the survey after 6 months. After the intervention, game jam participants did not have better intentions of culturally safe behaviour than did participants in the reference group (difference in means: 0.08 95% CI − 0.05 to 0.23); both groups had an improvement in this outcome. Multivariate analysis adjusted by clusters confirmed that game jam learning was associated with higher transcultural self-efficacy immediately after the intervention (wt OR 2.03 cl adj 95% CI 1.25–3.30).

**Conclusions:**

Game jam learning improved cultural safety intentions of Colombian medical students to a similar degree as did a carefully designed lecture and interactive workshop. The game jam was also associated with positive change in participant transcultural self-efficacy. We encourage further research to explore the impact of cultural safety training on patient-related outcomes. Our experience could inform initiatives to introduce cultural safety training in other multicultural settings.

**Trial registration:**

Registered on ISRCTN registry on July 18th 2019. Registration number: ISRCTN14261595.

**Supplementary Information:**

The online version contains supplementary material available at 10.1186/s12909-021-02545-7.

## Background

In Colombia, more than 40% of the population turn to traditional and cultural health practices, [1] but public and private institutions promote health services grounded in the Western biomedical model. Medical students are not trained to acknowledge and address intercultural tensions that arise in clinical practice. These tensions hinder patient access to effective health services, [2, 3] especially for those who use traditional health practices [4]. Cultural safety training of Colombian health professionals could address intercultural tensions, thus improving the access of patients from non-dominant cultures to health services.

Cultural safety is “a space that is spiritually, socially, emotionally and physically safe for people; where there is no assault, challenge or denial of their identity, of who they are, and what they need.”(p213, [5]) Irihapeti Ramsden, a Maori nurse, developed the concept to bridge the cultural divide between the Maori people and official health services in New Zealand [6]. A concept analysis of cultural safety [7] identified three foundations: equal partnership, active participation of patients from non-dominant cultures, and protection of cultural identity and well-being.

Cultural safety has gained attention because it offers a more respectful way to approach culture than cultural competence, the current standard approach [8]. Unlike cultural competence, cultural safety invites patients from non-dominant cultures to co-design and evaluate culturally safe health care [7, 9]. This participation in health care design also differentiates cultural safety from cultural humility, [10] another popular approach to cultural diversity in health care.

Observational studies suggest that cultural safety training may enhance respect for and acceptance of traditional and cultural health practices. It may also improve the relationships between health professionals and patients from non-dominant cultures, promote changes in knowledge, attitudes, self-confidence and behavior of health professionals, and lead to healthier outcomes [11]. There is a recognised lack of rigour in cultural safety assessment, however, and a need for formal randomised controlled trials (RCT) to evaluate cultural safety education [12].

Cultural safety is a well-established concept in New Zealand and Australia, but Canadian researchers and educators have only recently called for implementing cultural safety in healthcare practice [13]. There is little research on how best to apply this approach in medical education, [14] and on how health professionals can gain cultural safety skills [15]. There is also a pressing need to expand cultural safety initiatives to other culturally diverse settings, such as Latin American countries, and in non-Indigenous populations [7].

Cultural safety education of medical students is challenging. Contemporary medical training is overloaded, with little space to introduce new subjects. Educators might find cultural safety complicated to teach and medical students might perceive it to be dull or even unnecessary [16]. Going beyond simple knowledge acquisition, the educational experience of cultural safety must be *transformative* if it is to impact behavior [12]. Millennial students have novel ways of learning that include technology, creativity, and amusement [17, 18]. Game jams offer an engaging learning environment for this generation. Game jams are participatory events for attendees to create games in a time-constrained environment, typically 48 h [19]. The experience fosters learning through interacting with others, [20] an essential aspect of transformative learning [21]. Game jams have a positive impact on the performance of computing students, [19, 20] personal, interpersonal, and STEAM (science, technology, engineering, arts, and mathematics) skills, and game development skills [22].

The educational dimension of game jams is promising, but this research is still in its infancy [22]. To the best of our knowledge, the current literature reflects no game jam learning initiative with medical students, and no RCT has explored cultural safety in medical education. Our primary aim was thus to determine whether medical student participation in a game jam on cultural safety is more effective than a standard lesson in changing self-reported intended patient-oriented behavior. The secondary objective was to determine the impact of game jam learning on student confidence in their general transcultural skills.

## Methods

### Trial design

A parallel-group, two-arm RCT with 1:1 allocation compared game jam participation with a standard lesson plus an interactive workshop on cultural safety. The RCT addressed the question: *Among medical students and interns from University of La Sabana, compared with a standard lesson plus a workshop on cultural safety, does game jam participation result in improved student and intern self-reported intended behavior, and confidence in transcultural skills?*

We followed the CONSORT 2010 updated guidelines for reporting parallel group randomised trials [23] (Additional file 1: CONSORT 2010 checklist of the study). We registered our study on ISRCTN registry on July 18th, 2019 (registration number: ISRCTN14261595, [24]} and published the protocol of our study prior to completion of recruitment [25] (Additional file 2).

### Study setting and participants

*La Sabana*, in Chia municipality near Bogota, is a private university with 8926 undergraduate students. In July 2019, there were 956 medical students and 256 medical interns (*N* = 1212) enrolled in the Faculty of Medicine [26]. In Colombia, medical interns are undergraduate medical trainees who have completed their basic medical training and are undertaking one to 2 years of supervised medical practice in teaching hospitals. The inclusion criteria for this trial were being a medical student or medical intern at any level of training and giving written informed consent. The exclusion criteria were being underage or not wanting to take part in the study.

### Interventions

The trial intervention was a game jam to create a low-tech prototype of an educational game promoting cultural safety in medical education. Groups of five or six students or medical interns took part in a six-step game jam comprising: (a) preliminary lecture session (one hour); (b) opening ceremony; (c) game building (four hours); (d) game testing (one hour); (e) game refining (30 min); and (f) closing (one-and-a-half-hours).

Shortly before the intervention began, our academic partners at the University of *La Sabana* requested an interactive workshop, to foster problem-based and communicative learning, for the control group, rather than just a standard lesson. The reference group thus received a different intervention, beginning with a one-and-a-half-hour lecture on cultural safety. After the PowerPoint-based lesson, a six-hour interactive workshop focused on selected cultural safety readings. Groups of five or six students or medical interns followed a study guide based on the lecture and the readings, creating posters to display their responses to other students. The activity duration was the same (eight hours) in the game jam and reference groups.

Because during game jams participants typically devote most of their time to creating games, the game jam lecture was slightly shorter than the reference group lecture. Both lectures reflected a cultural safety curriculum previously co-designed with traditional medicine users, medical students, and cultural safety experts. A protocol of the study to co-design the curriculum is available, [27] and the results will be reported soon. To develop the curriculum, stakeholders responded to semi-structured questionnaires, and participated in focus groups and deliberative dialogue groups. A member-checking strategy shared the co-designed curriculum with the stakeholders, who modified and approved the final version. The curriculum has five learning objectives: (a) culturally unsafe practices: acknowledge the intercultural tensions in health care and its consequences; (b) cultural awareness: examine their own attitudes, beliefs, and values, and how they shape their professional practice; (c) cultural humility: listen and learn from their patients’ traditional practices; (d) cultural competence: describe and compare current pedagogical approaches to address cultural diversity in healthcare and their limitations; and (e) cultural safety: discuss with patients to agree on their treatment. We assessed acceptability of an early version of the curriculum in our pilot RCT, which will be reported soon.

A Colombian MD with a Master of Science in Epidemiology, 6 years of teaching experience, and 9 years of intercultural research experience led the intervention group activities. A Colombian MD with a Master of Public Health, 18 years of teaching experience and 20 years of intercultural research experience, led the reference group activities.

### Outcomes

To the best of our knowledge, there are no validated research instruments to measure cultural safety outcomes in medical trainees. Cultural safety training should go beyond mere knowledge acquisition to promote behavioral changes. The primary outcome of the trial was the students’ self-reported intention to change their patient-related behavior. This was measured by the response to the statement: *I will never be open to include my patients’ cultural beliefs and practices in the health decision-making process*. It corresponded to the Intention to **C**hange intermediate outcome of the CASCADA model of planned behavior, [28] which has been successfully used to explore dengue prevention behavior [29]. We assessed the students’ intended behavior instead of actual practice or action, which would have required follow-up of clinical practice over several years. Our primary concern was the sustained intention to change measured 6 months post-intervention.

A supplementary analysis using transitive closure [30] examined the primary outcome in the context of the CASCADA results chain of **C**onscious knowledge, **A**ttitude, **S**ubjective norm, Intention to **C**hange, **A**gency, and **D**iscussion related to cultural safety. The questions used to assess each component of the CASCADA model are available (Additional file 3).

Several authors state that cultural safety is preceded by generic cultural knowledge and skills. Brascoupé, for example, points out that cultural competence provides a foundation for cultural safety [31]. Ramsden sees cultural safety training as a dynamic process moving from cultural awareness to cultural sensitivity to cultural safety [3]. Following this rationale, our secondary outcome was students’ confidence in their general transcultural skills (transcultural self-efficacy).

We assessed our primary and secondary outcomes at baseline, immediately following the teaching session, and 6 months post-intervention. The 37-item instrument included three parts. The first part (11 items) explored sociodemographic characteristics. The second part (19 items) was based on the validated Likert-type Transcultural Self–Efficacy Tool — Multidisciplinary Healthcare Provider version (TSET–MHP) [32]. The third part (cultural safety), was a seven-item Likert-type local questionnaire based on our CASCADA variables and tested for validity and reliability in our pilot RCT. Participants responded to questions using mobile devices and SurveyMonkey.

### Sample size

Using the *pwr* package in R, [33] we estimated that a group size of 199 participants in the game jam group and 199 participants in the control group (sample size = 398) could detect an effect size of 0.25, with a two-sided alpha = 0.05 and a power = 0.8. Our pilot RCT found an effect size (Cohen’s *d*) of 0.25 between intervention and control arms after the teaching session. Because we observed considerable contamination between intervention and control groups in the pilot, 0.25 was a conservative estimate of effect size.

### Recruitment and randomisation

We contacted medical students and medical interns via email, using mailing lists from the University of *La Sabana,* to invite them to participate. We stratified randomization by student intended patient-oriented behavior at baseline to address a possible imbalance in cultural safety awareness between the intervention and control groups before the intervention. The results of the baseline survey stratified medical students into groups with low and high scores for cultural safety. Computerized randomization allocated the students equally (1:1) to intervention or control arms. The first author generated the allocation sequence, enrolled participants, and assigned them to intervention or control group.

Blinding is nearly impossible in education research RCTs, but participants were not aware of the allocation sequence or their group allocation until the start of the intervention. They only knew the auditorium they had to attend on the day of the intervention. Twenty facilitators prevented students from switching allocation status.

### Data analysis

We used an intention-to-treat approach for the primary and secondary analysis. The primary analysis used a *t*-test to assess the effect of the intervention on the primary outcome 6 months after the intervention. Additionally, probabilistic transitive closure explored the influence of the CASCADA results chain on the primary outcome. This approach, developed by Andersson and colleagues, [30] estimated the net influence of each element of the results chain on each other element, and the penultimate outcome, which was **D**iscussion [34].

The secondary analysis focused on the secondary outcome of transcultural self-efficacy. A paired *t*-test assessed within-group comparisons (baseline and post-intervention I and II) and a simple *t*-test assessed between-group comparisons (treated versus control post-intervention) of the students’ confidence (transcultural self-efficacy) in their general transcultural skills. Additionally, a simple *t*-test compared the mean difference in the mean between the baseline and the third timepoint between the intervention and control groups.

For statistically significant differences in the between-group comparisons, we conducted a multivariate analysis to adjust for baseline variables. These included sex, place of birth and residence, subsistence farmers in the family, socioeconomic level, traditional medicine use by the family or the participant, medicinal plants planted at home, level of training, age, and clustering (work group during the intervention or control activities).

The multivariate analysis relied on the Mantel-Haenszel approach adjusted for cluster. We repeated the analysis using generalized estimating equations (GEE) to cross-check our results. The Lamothe cluster-adjusted Mantel-Haenszel is a non-parametric approach that is simple to compute and does not require any assumptions for binomial data [35, 36]. GEE is not intended to model between-cluster variation but focuses on the within cluster similarity of the residuals [36, 37].

Sensitivity and subgroup analyses using the Lamothe cluster-adjusted Mantel-Haenszel procedure explored the effect of game jam learning on transcultural self-efficacy within different groups (such as those using or not using traditional medicine at baseline). All statistical tests were two-sided at the 0.05 level of significance. The Bonferroni method [38] adjusted the level of significance for the number of tests used to assess the primary and secondary outcomes. To determine the number of tests used, we considered the type of test (simple or paired) and outcome (primary or secondary).

### Ethics

This study embraced the bioethical principles proposed by the Council for International Organizations of Medical Sciences, [39] the Declaration of Helsinki, [40] the guidelines on conducting research in class from the University of Alberta, [41] and the Tri-Council Policy Statement [42].

The Institutional Review Board of the McGill’s Faculty of Medicine (approval number A05-B37-17B) and the Sub-committee for Research of the Faculty of Medicine at University of *La Sabana* (approval number 445) provided ethical clearance for the study. All participants signed written informed consent before proceeding with any research activity.

## Results

**Fig. 1:**
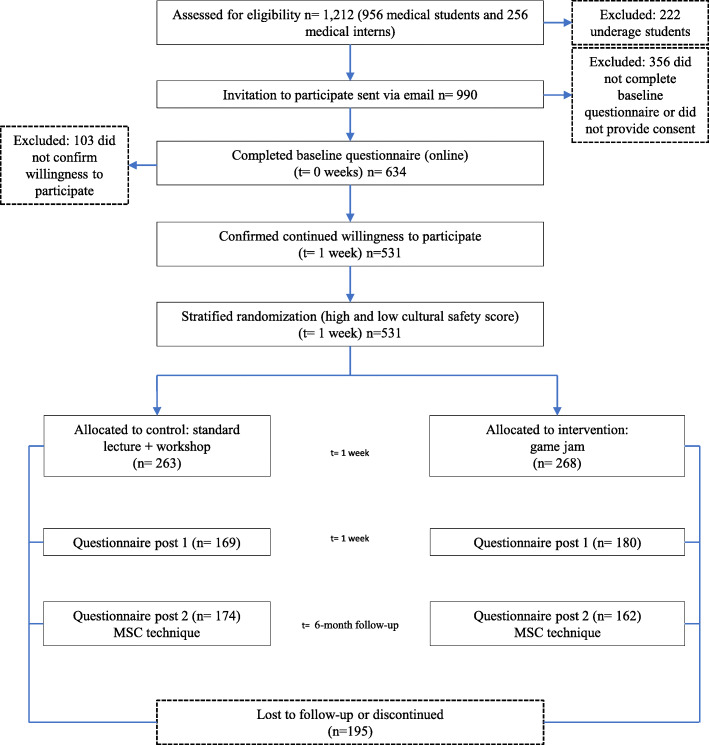
CONSORT flow diagram of the RCT

Some 531 students completed the baseline survey and were randomised; 347 students completed the second timepoint assessment, and 336 students completed the third timepoint assessment (Fig. 1). Of the 195 participants who were lost to follow-up, most completed the baseline survey and were randomised, but could not attend the study activities because of scheduling clashes. Additional file 4 is an attrition diagram [43] demonstrating the retention of participants over time.

The intervention and control groups were similar for all baseline sociodemographic characteristics (Table 1). Some 85.4% (229/268) and 78.7% (207/263) of the students’ families had used traditional medicine in the intervention and control group, respectively; this difference was marginally statistically significant. 61.2% (164/268) and 54.4% (143/263) of the participants had used traditional medicine in the intervention and control group (Table 1). This difference was not statistically significant at the 5% level.

**Table 1:** Baseline sociodemographic characteristics of the participants of the study

	Lesson and interactive workshop (***n*** = 263)	Game jam (***n*** = 268)	***p***-value
**Sex**	**n (%)**	**n (%)**	
Female	184 (70)	182 (67.9)	0.67
Male	78 (29.6)	85 (31.7)	0.67
Prefer not to say it	1 (0.4)	1 (0.4)	1
**Place of birth**			
Bogota	141 (53.6)	146 (54.5)	0.91
Colombia, another city	85 (32.3)	96 (35.8)	0.44
Venezuela	28 (10.6)	19 (7.1)	0.19
Another country	9 (3.5)	7 (2.6)	0.77
**Place of residency**			
Bogotá	176 (66.9)	169 (63.1)	0.4
Colombia, another city	87 (33.1)	99 (36.9)	0.4
**Family in rural settings**			
Yes	103 (39.1)	97 (36.2)	0.53
No	138 (52.4)	153 (57.1)	0.32
Do not know	22 (8.4)	18 (6.7)	0.57
**Socioeconomic level**			
One – lowest	3 (1.1)	5 (1.9)	0.74
Two	12 (4.6)	12 (4.5)	1
Three	44 (16.8)	55 (20.5)	0.31
Four	88 (33.5)	85 (31.7)	0.73
Five	65 (24.7)	67 (25)	1
Six – highest	40 (15.2)	34 (12.7)	0.47
Prefer not to say	3 (1.1)	2 (0.7)	0.98
Don’t know	8 (3)	8 (3)	1
**Family uses traditional medicine**			
Yes	207 (78.7)	229 (85.4)	0.055
No	34 (12.9)	24 (9)	0.18
Do not know	22 (8.4)	15 (5.6)	0.27
**Student uses traditional medicine**			
Yes	143 (54.4)	164 (61.2)	0.13
No	114 (43.3)	101 (37.7)	0.21
Do not know	6 (2.3)	3 (1.1)	0.48
**Medicinal plants planted at home**			
Yes	80 (30.4)	90 (33.6)	0.49
No	167 (63.5)	151 (56.3)	0.11
Do not know	16 (6.1)	27 (10.1)	0.12
**Education level**			
II and III semester (preclinical)	58 (22.1)	62 (23.1)	0.84
VI to XI (Clinical)	190 (72.2)	191 (71.3)	0.87
Medical intern	15 (5.7)	15 (5.6)	1
**Age in years**			
Min	18	18	1
Max	31	31	1
Mean (SD)	20.96 (1.9)	20.98 (1.9)	0.91

SD = Standard Deviation

Game jam participants did not have better intended culturally safe behaviour than did participants in the lesson and interactive workshop (difference in means: 0.08, 95% CI − 0.05 to 0.23). Game jam learning was superior to the more conventional learning in terms of transcultural self-efficacy immediately after the intervention (difference in means: 0.12, 95% CI 0.02 to 0.2), but not 6 months after the intervention (difference in means: 0.6, 95% CI − 0.06 to 0.11) (Table 2). We did not detect a statistically significant difference in the mean change between the baseline and the third timepoint between the intervention and control groups.

**Table 2:** Difference in the means of primary and secondary outcomes between intervention groups

Students’ self-reported intended patient-oriented behavior					
	Lesson + workshop	Game jam	Difference	95% CI	***n=***
Pre-intervention	4.39	4.39	0	− 0.1 to 0.1	531
Post-intervention 2	4.54	4.62	0.08	−0.05 to 0.23	336
**Transcultural self-efficacy**					
	**Lesson + workshop**	**Game jam**	**Difference**	**95% CI**	***n=***
Pre-intervention	4	3.96	−0.04	−1 to 0.02	531
Post-intervention 1	4.18	4.3	0.12	**0.02 to 0.2**	347
Post-intervention 2	4.13	4.15	0.02	−0.06 to 0.11	331

* Significant differences are shown in bold font

Probabilistic transitive closure of the CASCADA results chain showed good progression to the last outcome (**D**iscussion) with no blocks in the game jam and control groups (net influence of 13.75 and 13.34 respectively). The baseline values favoured the control group (net influence of 5.12 in the intervention arm and 9.7 in the control arm). The change from baseline to the 6 month assessment was larger in the intervention group.

The game jam and standard lesson plus workshop both had positive and similar effects on the primary and secondary outcomes (Table 3). We used the Bonferroni correction to adjust the alpha level of the simple and paired t-test exploring differences in the primary and secondary outcomes. All non-adjusted significant associations remained significant after adjustment.

**Table 3:** Difference in primary and secondary outcomes within intervention groups

Students’ self-reported intended patient-oriented behavior					
	Pre-intervention	Post-intervention 2	Difference	95% CI	***n=***
Total	4.37	4.58	0.25	**0.11 to 0.29**	336
Lesson + workshop	4.33	4.54	0.21	**0.07 to 0.33**	174
Game jam	4.41	4.62	0.21	**0.09 to 0.32**	162
**Transcultural self-efficacy**					
	**Pre-intervention**	**Post-intervention 1**	**Difference**	**95% CI**	***n=***
Total	3.97	4.24	0.27	**0.22 to 0.30**	347
Lesson + workshop	3.99	4.18	0.19	**0.13 to 0.24**	167
Game jam	3.96	4.3	0.34	**0.28 to 0.4**	180
	**Pre-intervention**	**Post-intervention 2**	**Difference**	**95% CI**	***n=***
Total	3.99	4.14	0.15	**0.1 to 0.19**	328
Lesson + workshop	3.99	4.13	0.14	**0.07 to 0.19**	170
Game jam	3.99	4.15	0.16	**0.08 to 0.23**	158
	**Post-intervention 1**	**Post-intervention 2**	**Difference**	**95% CI**	***n=***
Total	4.24	4.17	- 0.007	**−0.02 to − 0.13**	259
Lesson + workshop	4.16	4.16	0	−0.05 to 0.06	128
Game jam	4.34	4.18	- 0.16	**−0.07 to − 0.23**	131

* Significant differences are shown in bold font

The multivariate analysis using the Mantel-Haenszel approach adjusted by clusters confirmed that game jam learning and traditional medicine use reported by students was associated with higher transcultural self-efficacy immediately after the intervention (wt OR 2.03 cl adj 95% CI 1.25–3.30 and wt OR 1.98 cl adj 95% CI 1.11–2.84, respectively). GEE confirmed that game jam learning was associated with higher transcultural self-efficacy immediately after the intervention (adj OR = 2.09 95% CI 1.22–3.60).

**Fig. 2:**
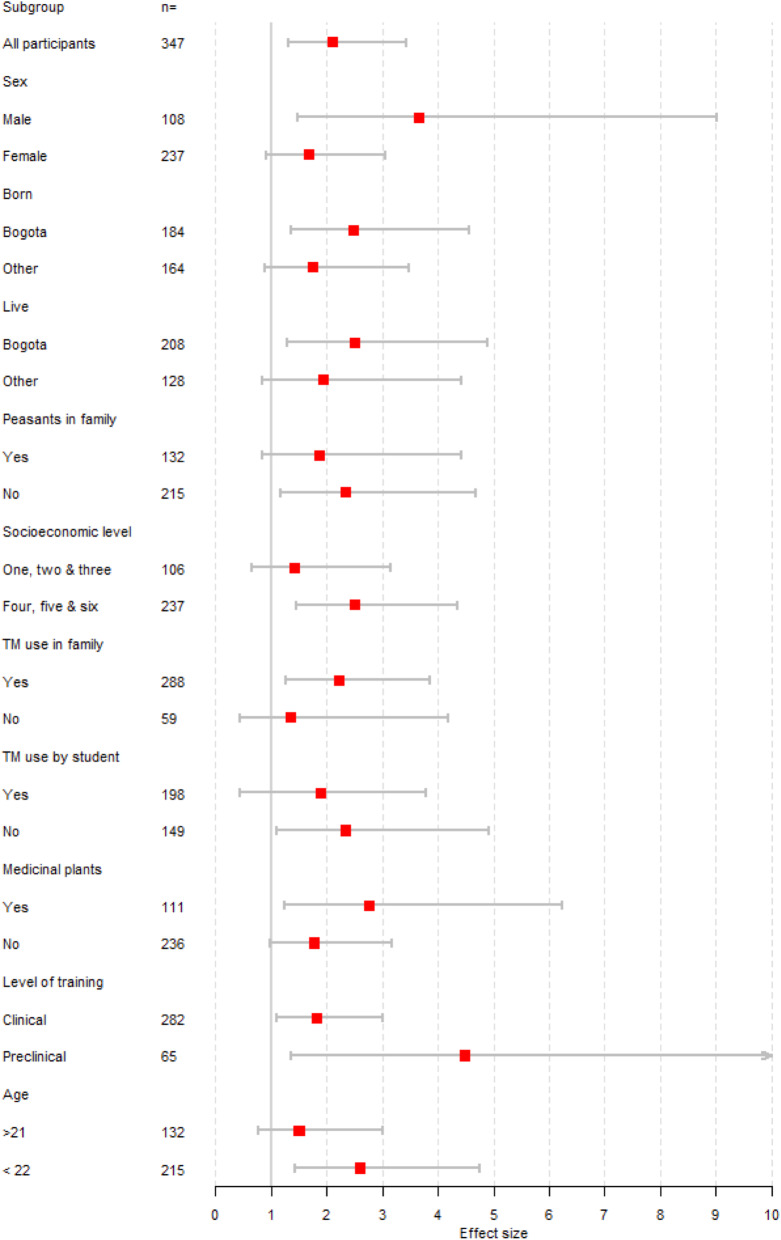
Game jam learning effect on transcultural self-efficacy according to subgroup

Although not statistically significant at the 5% level, sensitivity analysis suggested a stronger effect of game jam learning on transcultural self-efficacy among students who were male, born and living in Bogota, with no subsistence farmers in the family, and from a higher socioeconomic level (Fig. 2).

## Discussion

Separately, both the game jam and the lesson plus interactive workshop had positive impacts on the primary and secondary outcomes. Both groups received training on the same key elements of cultural safety based on a rigorous curriculum that Colombian stakeholders co-created through a sequential-consensual qualitative study [27]. Notwithstanding the original hypothesis, our study provides evidence of the effectiveness of cultural safety training based on our co-designed curriculum. 

In this trial, game jam learning changed cultural safety intention, but it was not superior to more conventional learning approaches. We are aware of several potential explanations for this. First, contamination is a well-known concern of parallel-group RCTs in education and could have influenced the results of the primary outcome comparison. During the 6 months before the third timepoint, individuals who received the intervention could have *leaked information* about their experience in the game jam, influencing results in the control group. This would have reduced the measured impact of the intervention, making it more difficult to find a significant difference between the groups [44]. Second, a Hawthorne effect, triggered by awareness of observation and assessment, [45] could have influenced both the intervention and control group participants, thus decreasing the opportunity to detect a significant difference between the groups. Third, in designing the study we used the World Health Organization estimate that 40% of Colombians seek care in traditional and cultural health practices [1]. In the pre-intervention baseline, however, we found more than twice that proportion came from families that used traditional medicine (Table 1). This was associated with quite high initial levels of intended culturally safe behavior and reduced the potential for improvement after the intervention.

Fourth, and probably most important, the academic partners at the University of *La Sabana* requested an interactive complementary activity for the control group in the form of a workshop, to foster problem-based and communicative learning, two of the main elements of transformative learning [46]. This turned the “control” into an intervention in its own right. A professor with substantial intercultural experience led the control group activities, resulting in an unusually strong learning experience in the control group.

We believe the game jam learning would show a bigger impact if contrasted with a standard lesson on the subject without components of transformative learning. Medical education studies often use no control group and, when they use one, control participants receive no training on the subject of interest [47]. Cook summarized four meta-analyses [48–51] with over 750 studies comparing various forms of education against no intervention. Almost all these studies favoured the training group for outcomes of knowledge, skills, and behaviours, [47] confirming that an “educational placebo-controlled trial has very limited value.” [52] Similar to clinical research, where placebo-controlled research is often unethical, recent trends in medical education research advocate for comparative effectiveness, where control groups receive active interventions [53]. Despite such a study being harder to design and conduct, in our trial we opted to assess comparative effectiveness. Studies using this approach require much larger sample sizes than placebo-controlled trials since the expected effect size is reduced [47]. The effective sample size for our analysis of the primary outcome was smaller than the calculated sample size.

Our multivariate analysis confirmed that game jam learning was associated with higher transcultural self-efficacy immediately after the intervention. A recent game jam promoted self-discovery, reflections on identity, and support for the cultural identity of the Sami people in Finland [54]. Ramsden suggested that cultural safety is a continuum rather than a fixed state, with prior steps such as cultural awareness and cultural sensitivity [55]. Our co-designed curriculum, which informed the intervention and control learning activities, also included prior steps before cultural safety, such as cultural risk, awareness, humility, and competence. These prior steps might be more easily identified by the TSET, which detects changes in knowledge, attitudes, and skills of cultural competence [56]. As opposed to the section of the questionnaire that explored changes in the primary outcome of cultural safety intention, the TSET is a widely validated and used research instrument, [57] which could have facilitated detecting a significant effect on the secondary outcome of transcultural self-efficacy.

### Limitations

Reproducibility of educational interventions is hard to ensure due to the well-known “teacher effect”, with results of teaching interventions depending on the abilities and skills of individual instructors [58]. We followed recommendations to maximize the reproducibility and generalisability of our intervention, [59] like describing the intervention in detail to allow reproducibility and scrutiny in the future and providing the background of the instructors involved in the study activities. We recognize the experience of the reference group instructor influenced results in this group, effectively reducing the difference between this group and the game jam group.

We did not achieve and retain our intended sample size of 398 trainees, and this reduced the power of the study to detect differences, especially at the second and third timepoints. Future medical education RCTs should use more robust logistical methods to ensure the desired sample size is achieved and retained.

There is merit in using patient-related outcomes such as evaluations of care received, health outcomes, and health behaviors to assess the effect of cultural safety interventions [60]. Given the time available for the study and the complexity of assessing patient-related outcomes in medical student education, we used education-related outcomes based on a theory of planned behavior. We included a qualitative understanding through the Most Significant Change evaluation (to be reported separately). Future cultural safety training initiatives should include patient-related outcomes to determine the impact of the interventions directly on the patients and their communities.

Finally, our findings are specific to the Colombian cultural context. In other settings, where traditional health practices are not widespread, it might be necessary to provide cultural safety training based on other cultural characteristics. For example, there are cultural safety experiences reported in Canada with Amish and Low German Mennonites [61].

## Conclusion

Game jam learning improved cultural safety intentions of Colombian medical students to a similar degree as did a carefully designed lecture and workshop. The game jam was also associated with positive change in participant transcultural self-efficacy. Potential contamination and a strong control learning activity with an experienced instructor and elements of transformative learning likely precluded the detection of a significant difference in the primary outcome.

This is the first published RCT of cultural safety in medical education and one of few attempts to apply cultural safety to non-Indigenous yet culturally rich settings. Our research yielded key lessons applicable to other multicultural countries requiring cultural safety training in medical education. We encourage further research to explore the impact of cultural safety training on population health, ideally using patient-related outcomes and designs that are less prone to contamination, such as cluster RCTs.

**Protocol:** The protocol of this RCT was accepted for publication before completion of recruitment [25].

## Supplementary Information

**Additional file 1.** CONSORT checklist of information to include when reporting an RCT– filled CONSORT checklist.

**Additional file 2.** Impact of Co-Designed Game Learning on Cultural Safety in Colombian Medical Education: Protocol for a Randomized Controlled Trial - Protocol of our study published in the Journal of Medical Internet Research – Research protocols.

**Additional file 3.** Questions used to assess each component of the CASCADA model – questions used in the quantitative questionnaire.

**Additional file 4.** Attrition diagram of the study - attrition diagram of the study.

## Data Availability

The datasets used and/or analysed during the current study will be available from the corresponding author on reasonable request.

## Abbreviations

STEAM: Science, technology, engineering, arts, and mathematics
RCT: Randomised Controlled Trial
CONSORT: Consolidated Standards of Reporting Trials
CASCADA: **C**onscious knowledge, **A**ttitudes, **S**ubjective norms, **C**hange intention, sense of **A**gency, socialization/**D**iscussion, and behavior change/**A**ction
TSET–MHP: Transcultural Self–Efficacy Tool — Multidisciplinary Healthcare Provider version
SD: Standard Deviation
GEE: Generalized estimating equation
