# Supplementary material for: Impact of game jam learning about cultural safety in Colombian medical education: a randomised controlled trial
Source: BMC Med Educ. 2021 Feb 25;21:132. doi: 10.1186/s12909-021-02545-7 (PMC7905593; doi:10.1186/s12909-021-02545-7)
Supplement: Supplementary file 1 — Additional file 1. CONSORT checklist of information to include when reporting an RCT– filled CONSORT checklist. [file 12909_2021_2545_MOESM1_ESM.docx]

**Impact of Game Jam Learning about Cultural Safety in Colombian Medical Education: a Randomised Controlled Trial**

**Authors**

Juan Pimentel, Anne Cockcroft, and Neil Andersson

# **Additional file 1. Reporting checklist for randomised trial.**

Based on the CONSORT guidelines.

## Instructions to authors

Complete this checklist by entering the page numbers from your manuscript where readers will find each of the items listed below.

Your article may not currently address all the items on the checklist. Please modify your text to include the missing information. If you are certain that an item does not apply, please write "n/a" and provide a short explanation.

Upload your completed checklist as an extra file when you submit to a journal.

In your methods section, say that you used the CONSORTreporting guidelines, and cite them as:

Schulz KF, Altman DG, Moher D, for the CONSORT Group. CONSORT 2010 Statement: updated guidelines for reporting parallel group randomised trials

|  |  | Reporting Item | Page Number |
| --- | --- | --- | --- |
| **Title and Abstract** |  |  |  |
| Title | [#1a](https://www.goodreports.org/consort/info/#1a) | Identification as a randomized trial in the title. | 1 |
| Abstract | [#1b](https://www.goodreports.org/consort/info/#1b) | Structured summary of trial design, methods, results, and conclusions | 2 & 3 (see our completed CONSORT for Abstracts checklist) |
| **Introduction** |  |  |  |
| Background and objectives | [#2a](https://www.goodreports.org/consort/info/#2a) | Scientific background and explanation of rationale | 4 & 5 |
| Background and objectives | [#2b](https://www.goodreports.org/consort/info/#2b) | Specific objectives or hypothesis | 5 & 6 |
| **Methods** |  |  |  |
| Trial design | [#3a](https://www.goodreports.org/consort/info/#3a) | Description of trial design (such as parallel, factorial) including allocation ratio. | 6 - Trial design |
| Trial design | [#3b](https://www.goodreports.org/consort/info/#3b) | Important changes to methods after trial commencement (such as eligibility criteria), with reasons | 7 - Interventions |
| Participants | [#4a](https://www.goodreports.org/consort/info/#4a) | Eligibility criteria for participants | 6 - Study setting and participants |
| Participants | [#4b](https://www.goodreports.org/consort/info/#4b) | Settings and locations where the data were collected | 6 - Study setting and participants |
| Interventions | [#5](https://www.goodreports.org/consort/info/#5) | The experimental and control interventions for each group with sufficient details to allow replication, including how and when they were actually administered | 7 - Interventions |
| Outcomes | [#6a](https://www.goodreports.org/consort/info/#6a) | Completely defined prespecified primary and secondary outcome measures, including how and when they were assessed | 8 - Outcomes |
| Sample size | [#7a](https://www.goodreports.org/consort/info/#7a) | How sample size was determined. | 9 - Sample size |
| Sample size | [#7b](https://www.goodreports.org/consort/info/#7b) | When applicable, explanation of any interim analyses and stopping guidelines | N/A |
| Randomization - Sequence generation | [#8a](https://www.goodreports.org/consort/info/#8a) | Method used to generate the random allocation sequence. |  |
| 9 - Recruitment and randomisation |  |  |  |
| Randomization - Sequence generation | [#8b](https://www.goodreports.org/consort/info/#8b) | Type of randomization; details of any restriction (such as blocking and block size) |  |
| 9 - Recruitment and randomisation |  |  |  |
| Randomization - Allocation concealment mechanism | [#9](https://www.goodreports.org/consort/info/#9) | Mechanism used to implement the random allocation sequence (such as sequentially numbered containers), describing any steps taken to conceal the sequence until interventions were assigned | 9 - Recruitment and randomisation |
| Randomization - Implementation | [#10](https://www.goodreports.org/consort/info/#10) | Who generated the allocation sequence, who enrolled participants, and who assigned participants to interventions | 9 - Recruitment and randomisation |
| Blinding | [#11a](https://www.goodreports.org/consort/info/#11a) | If done, who was blinded after assignment to interventions (for example, participants, care providers, those assessing outcomes) and how. | 9 - Recruitment and randomisation |
| Blinding | [#11b](https://www.goodreports.org/consort/info/#11b) | If relevant, description of the similarity of interventions | N/A |
| Statistical methods | [#12a](https://www.goodreports.org/consort/info/#12a) | Statistical methods used to compare groups for primary and secondary outcomes | 9 & 10 - Data analysis |
| Statistical methods | [#12b](https://www.goodreports.org/consort/info/#12b) | Methods for additional analyses, such as subgroup analyses and adjusted analyses | 9 & 10 - Data analysis |
| Outcomes | [#6b](https://www.goodreports.org/consort/info/#6b) | Any changes to trial outcomes after the trial commenced, with reasons | N/A |
| **Results** |  |  |  |
| Participant flow diagram (strongly recommended) | [#13a](https://www.goodreports.org/consort/info/#13a) | For each group, the numbers of participants who were randomly assigned, received intended treatment, and were analysed for the primary outcome | Figure 1 CONSORT flow diagram of the RCT |
| Participant flow | [#13b](https://www.goodreports.org/consort/info/#13b) | For each group, losses and exclusions after randomization, together with reason | Figure 1 CONSORT flow diagram of the RCT and Additional file 2 is an attrition diagram |
| Recruitment | [#14a](https://www.goodreports.org/consort/info/#14a) | Dates defining the periods of recruitment and follow-up | Figure 1 CONSORT flow diagram of the RCT |
| Recruitment | [#14b](https://www.goodreports.org/consort/info/#14b) | Why the trial ended or was stopped | Figure 1 CONSORT flow diagram of the RCT |
| Baseline data | [#15](https://www.goodreports.org/consort/info/#15) | A table showing baseline demographic and clinical characteristics for each group | Table 1. Baseline sociodemographic characteristics of the participants of the study |
| Numbers analysed | [#16](https://www.goodreports.org/consort/info/#16) | For each group, number of participants (denominator) included in each analysis and whether the analysis was by original assigned groups | Tables 2 & 3 |
| Outcomes and estimation | [#17a](https://www.goodreports.org/consort/info/#17a) | For each primary and secondary outcome, results for each group, and the estimated effect size and its precision (such as 95% confidence interval) | Tables 2 & 3 |
| Outcomes and estimation | [#17b](https://www.goodreports.org/consort/info/#17b) | For binary outcomes, presentation of both absolute and relative effect sizes is recommended | N/A |
| Ancillary analyses | [#18](https://www.goodreports.org/consort/info/#18) | Results of any other analyses performed, including subgroup analyses and adjusted analyses, distinguishing pre-specified from exploratory | 16 & 17 |
| Harms | [#19](https://www.goodreports.org/consort/info/#19) | All important harms or unintended effects in each group (For specific guidance see CONSORT for harms) | N/A - not a clinical trial |
| **Discussion** |  |  |  |
| Limitations | [#20](https://www.goodreports.org/consort/info/#20) | Trial limitations, addressing sources of potential bias, imprecision, and, if relevant, multiplicity of analyses | 20 & 21 - Limitations |
| Interpretation | [#22](https://www.goodreports.org/consort/info/#22) | Interpretation consistent with results, balancing benefits and harms, and considering other relevant evidence | 18 to 20 |
| Registration | [#23](https://www.goodreports.org/consort/info/#23) | Registration number and name of trial registry | 3 - Trial registration |
| **Other Information** |  |  |  |
| Protocol | [#24](https://www.goodreports.org/consort/info/#24) | Where the full trial protocol can be accessed, if available | 6 - Trial design |
| Funding | [#25](https://www.goodreports.org/consort/info/#25) | Sources of funding and other support (such as supply of drugs), role of funders | 23 - Funding |

Notes:

- 1b: 2 & 3 (see our completed CONSORT for Abstracts checklist)
- 3a: 6 - Trial design
- 3b: 7 - Interventions
- 4a: 6 - Study setting and participants
- 4b: 6 - Study setting and participants
- 5: 7 - Interventions
- 6a: 8 - Outcomes
- 7a: 9 - Sample size
- 8a: 9 - Recruitment and randomisation
- 8b: 9 - Recruitment and randomisation
- 9: 9 - Recruitment and randomisation
- 10: 9 - Recruitment and randomisation
- 11a: 9 - Recruitment and randomisation
- 12a: 9 & 10 - Data analysis
- 12b: 9 & 10 - Data analysis
- 13a: Figure 1 CONSORT flow diagram of the RCT
- 13b: Figure 1 CONSORT flow diagram of the RCT and Additional file 2 is an attrition diagram
- 14a: Figure 1 CONSORT flow diagram of the RCT
- 14b: Figure 1 CONSORT flow diagram of the RCT
- 15: Table 1. Baseline sociodemographic characteristics of the participants of the study
- 16: Tables 2 & 3
- 17a: Tables 2 & 3
- 19: N/A - not a clinical trial
- 20: 20 & 21 - Limitations
- 23: 3 - Trial registration
- 24: 6 - Trial design
- 25: 23 - Funding The CONSORT checklist is distributed under the terms of the Creative Commons Attribution License CC-BY. This checklist was completed on 26. August 2020 using <https://www.goodreports.org/>, a tool made by the [EQUATOR Network](https://www.equator-network.org) in collaboration with [Penelope.ai](https://www.penelope.ai)

# **Reporting checklist for randomised trial abstract.**

Based on the CONSORT for Abstracts guidelines.

## Instructions to authors

Complete this checklist by entering the page numbers from your manuscript where readers will find each of the items listed below.

Your article may not currently address all the items on the checklist. Please modify your text to include the missing information. If you are certain that an item does not apply, please write "n/a" and provide a short explanation.

Upload your completed checklist as an extra file when you submit to a journal.

In your methods section, say that you used the CONSORT for Abstractsreporting guidelines, and cite them as:

Hopewell S, Clarke M, Moher D, Wager E, Middleton P, Altman DG, Schulz KF and the CONSORT Group (2008) CONSORT for reporting randomised trials in journal and conference abstracts. Lancet: 371:281-283. PMID: 18221781

|  |  | Reporting Item | Page Number |
| --- | --- | --- | --- |
| **Title and study details** |  |  |  |
| Title | [#1](https://www.goodreports.org/consort-for-abstracts/info/#1) | Identification of the study as randomized | 1 |
| Authors | [#2](https://www.goodreports.org/consort-for-abstracts/info/#2) | Contact details for the corresponding author. | 1 |
| Trial Design | [#3](https://www.goodreports.org/consort-for-abstracts/info/#3) | Description of the trial design. | 2 - Methods |
| **Abstract subsection: Methods** |  |  |  |
| Participants | [#4a](https://www.goodreports.org/consort-for-abstracts/info/#4a) | Eligibility criteria for participants and the settings where the data were collected. | 2 - Methods |
| Participants | [#4](https://www.goodreports.org/consort-for-abstracts/info/#4) | Eligibility criteria for participants and the settings where the data were collected | 2 - Methods |
| Interventions | [#5](https://www.goodreports.org/consort-for-abstracts/info/#5) | Interventions intended for each group. | 2 - Methods |
| Objective | [#6](https://www.goodreports.org/consort-for-abstracts/info/#6) | Specific objective or hypothesis. | 2 - Background |
| Outcome | [#7](https://www.goodreports.org/consort-for-abstracts/info/#7) | Clearly defined primary outcome for this report. | 2 - Background |
| Randomization | [#8](https://www.goodreports.org/consort-for-abstracts/info/#8) | How participants were allocated to interventions. | 2 - Methods |
| Blinding (Masking) | [#9](https://www.goodreports.org/consort-for-abstracts/info/#9) | Whether or not participants, caregivers, and those assessing the outcomes were blinded to group assignment. | 2 - Methods |
| **Abstract subsection: Results** |  |  |  |
| Numbers Randomized | [#10](https://www.goodreports.org/consort-for-abstracts/info/#10) | Number of participants randomized to each group. | 2 - Results |
| Recruitment | [#11](https://www.goodreports.org/consort-for-abstracts/info/#11) | Trial status | 2 - Results |
| Numbers Analysed | [#12](https://www.goodreports.org/consort-for-abstracts/info/#12) | Number of participants analysed in each group. | 2 - Results |
| Outcome | [#13](https://www.goodreports.org/consort-for-abstracts/info/#13) | For the primary outcome, a result for each group and the estimated effect size and its precision. | 2 - Results |
| Harms | [#14](https://www.goodreports.org/consort-for-abstracts/info/#14) | Important adverse events or side effects. | N/A - not a clinical trial |
| **Abstract subsection: Conclusions** |  |  |  |
| Conclusions | [#15](https://www.goodreports.org/consort-for-abstracts/info/#15) | General interpretation of the results. | 3 - Conclusions |
| **Trial Registration** |  |  |  |
| Trial Registration | [#16](https://www.goodreports.org/consort-for-abstracts/info/#16) | Registration number and name of trial register. | 3 - Trial registration |
| **Funding** |  |  |  |
| Funding | [#17](https://www.goodreports.org/consort-for-abstracts/info/#17) | Source of funding. | 23 - Funding |

Notes:

- 4a: 2 - Methods
- 3: 2 - Methods
- 4: 2 - Methods
- 5: 2 - Methods
- 6: 2 - Background
- 7: 2 - Background
- 8: 2 - Methods
- 9: 2 - Methods
- 10: 2 - Results
- 11: 2 - Results
- 12: 2 - Results
- 13: 2 - Results
- 14: N/A - not a clinical trial
- 15: 3 - Conclusions
- 16: 3 - Trial registration
- 17: 23 - Funding This checklist was completed on 26. August 2020 using <https://www.goodreports.org/>, a tool made by the [EQUATOR Network](https://www.equator-network.org) in collaboration with [Penelope.ai](https://www.penelope.ai)
