# Supplementary material for: Impact of game jam learning about cultural safety in Colombian medical education: a randomised controlled trial
Source: BMC Med Educ. 2021 Feb 25;21:132. doi: 10.1186/s12909-021-02545-7 (PMC7905593; doi:10.1186/s12909-021-02545-7)
Supplement: Supplementary file 2 — Additional file 2. Impact of Co-Designed Game Learning on Cultural Safety in Colombian Medical Education: Protocol for a Randomized Controlled Trial - Protocol of our study published in the Journal of Medical Internet Research – Research protocols. [file 12909_2021_2545_MOESM2_ESM.docx]

**Impact of Game Jam Learning about Cultural Safety in Colombian Medical Education: a Randomised Controlled Trial**

**Authors**

Juan Pimentel, Anne Cockcroft, and Neil Andersson

**Additional file 2. Impact of Co-Designed Game Learning on Cultural Safety in Colombian Medical Education: Protocol for a Randomized Controlled Trial**

Our study protocol has been peer-reviewed and it was published prior to completion of recruitment:

Pimentel J, Cockcroft A, Andersson N. Impact of Co-Designed Game Learning on Cultural Safety in Colombian Medical Education: Protocol for a Randomized Controlled Trial. JMIR Res Protoc. 2020;9:e17297.

The protocol is available: <https://www.researchprotocols.org/2020/8/e17297/pdf>
