# Supplementary material for: Impact of game jam learning about cultural safety in Colombian medical education: a randomised controlled trial
Source: BMC Med Educ. 2021 Feb 25;21:132. doi: 10.1186/s12909-021-02545-7 (PMC7905593; doi:10.1186/s12909-021-02545-7)
Supplement: Supplementary file 3 — Additional file 3. Questions used to assess each component of the CASCADA model – questions used in the quantitative questionnaire. [file 12909_2021_2545_MOESM3_ESM.docx]

**Impact of Game Jam Learning about Cultural Safety in Colombian Medical Education: a Randomised Controlled Trial**

**Authors**

Juan Pimentel, Anne Cockcroft, and Neil Andersson

**Additional file 3. Questions used to assess each component of the CASCADA model**

The CASCADA (conscious knowledge, attitudes, subjective norms, change intention, sense of agency, discussion, and behavior/action) model of planned behavior[23] included the following variables:

| **Component** | **Statement** |
| --- | --- |
| ***C***onscious knowledge | “I consider the cultural beliefs of my patients is not important for health decision-making” |
| ***A***ttitude | “It is not worth considering the cultural beliefs of my patients to improve their health.” |
| ***S***ubjective norms | “Although many physicians disapprove cultural beliefs, I think that these beliefs could improve my patients’ health.” |
| Intention to ***c***hange | “I will never be open to include my patients’ cultural beliefs and practices in the health decision-making process.” |
| ***A***gency | “I feel prepared with the knowledge and skills to prudently incorporate my patients' cultural practices in the health decision-making process.” |
| ***D***iscussion | “I will discuss cultural safety with other students and physicians so they can prudently incorporate their patients' cultural practices in the health decision-making process.” |
