# Supplementary material for: Impact of game jam learning about cultural safety in Colombian medical education: a randomised controlled trial
Source: BMC Med Educ. 2021 Feb 25;21:132. doi: 10.1186/s12909-021-02545-7 (PMC7905593; doi:10.1186/s12909-021-02545-7)
Supplement: Supplementary file 4 — Additional file 4. Attrition diagram of the study - attrition diagram of the study. [file 12909_2021_2545_MOESM4_ESM.docx]

**Impact of Game Jam Learning about Cultural Safety in Colombian Medical Education: a Randomised Controlled Trial**

**Authors**

Juan Pimentel, Anne Cockcroft, and Neil Andersson

**Additional file 4. Attrition diagram of the study**

|  | **Intervention** | **Control** | **Total** |
| --- | --- | --- | --- |
| **Baseline** | 268 | 263 | 531 |
| **Second timepoint** | 180 | 169 | 349 |
| **Third timepoint** | 162 | 174 | 336 |
